# 

**Published:** 1893-07

**Authors:** 


					﻿POPULAR ERRORS IN MEDICINE!.
By JAMES H. DIXON, M. D.
io cts. a copy.
JULY, 1893.
$1.00 per year.
HAIJ A*
JOVRNALJ
HEALTH
ESTABLISHED 1854
Vol. XL
No. 7.
OFFICE, 23 PARK ROW.
Entered as Second-Class Matter at the Post-Office, New York.
				

